# Therapeutic targeting of PFKFB3 and PFKFB4 in multiple myeloma cells under hypoxic conditions

**DOI:** 10.1186/s40364-022-00376-2

**Published:** 2022-05-16

**Authors:** Seiichi Okabe, Yuko Tanaka, Akihiko Gotoh

**Affiliations:** Department of Hematology, Tokyo Medical University, 6-7-1 Nishi-shinjuku, Shinjuku-ku, Tokyo, 160-0023 Japan

**Keywords:** Hypoxia, Multiple myeloma, PFKFB, Proteasome inhibitor

## Abstract

The treatment of multiple myeloma (MM) patients has been dramatically changed by the introduction of new agents; however, many patients relapse. Hypoxia is a critical component of the bone-marrow microenvironment. 6-Phosphofructo-2-kinase/fructose-2,6-bisphosphatase (PFKFB) is responsible for maintaining cellular levels of fructose-2,6-bisphosphate, which regulates glycolysis. We found that the gene expressions of *PFKFB3* and *PFKFB4* were elevated under hypoxic conditions. Treatments with the PFKFB3 inhibitor, PFK158, and PFKFB4 inhibitor, 5MPN, were found to inhibit the growth of myeloma cells. The combined treatment of myeloma cells with carfilzomib and PFK158 or 5MPN was more cytotoxic than either drug alone. Caspase 3/7 activity and cellular cytotoxicity were also increased. In addition, the combined treatment was effective in the bortezomib-resistant cell line. Our data also suggest that administration of PFKFB3 and PFKFB4 inhibitors may be a powerful strategy against myeloma cells and to enhance the cytotoxic effects of proteasome inhibitors in hypoxic conditions.

**Supplementary Information:**

The online version contains supplementary material available at 10.1186/s40364-022-00376-2.

To the editors:

Multiple myeloma (MM) is a malignancy of terminally differentiated plasma cells in the bone marrow [1]. Therapeutic strategies for MM have dramatically changed after the introduction of bortezomib. However, acquisition of resistance causes a relapse of the disease in many myeloma patients, even after bortezomib treatment [2]. Thus, the clinical management of MM patients to improve their survival rates remains a challenge. Bone marrow is a tissue with a limited oxygen supply [3]. Fructose-2,6-bisphosphate accelerates glycolytic flux by allosterically activating 6-phosphofructo-1-kinase. Fructose-2,6-bisphosphate is generated and degraded by the bifunctional enzyme 6-phosphofructo-2-kinase/fructose-2,6-bisphosphatase (PFK-2/FBPase-2) [4]. There are four major PFK-2/FBPase-2 isozymes in vertebrates, 6-phosphofructo-2-kinase/fructose-2,6-bisphosphatase 1–4 (PFKFB-1–4), which are encoded by four different genes (*PFKFB1*, *PFKFB2*, *PFKFB3*, and *PFKFB4*) [5]. Therefore, we investigated whether hypoxia affects metabolic change in myeloma cells and evaluated the activity of the second-generation proteasome inhibitor, carfilzomib, using MM cell lines including those with bortezomib resistance.

The gene expressions of *PFKFB3* and *PFKFB4*, but not those of *PFKFB1* and *PFKFB2*, were higher under hypoxic conditions (1% O_2_) than in the normoxic samples (GSE80140) (Fig. 1A, Supplemental Fig. 1A). The protein expressions of PFKFB3 and PFKFB4 were increased (Fig. 1B, Supplemental Fig. 1B, C). The expression of phospho-p38 MAPK was increased by hypoxia. HIF1α confers resistance to conventional therapies via several signaling pathways, including apoptosis and mitochondrial activity [6]. HIF1α is beneficial for glycolysis and lactic acid production [6]. The protein expressions of HIF1α and PFKFB3 increased after 6 to 24 h under hypoxic conditions. In contrast, PFKFB4 expression was increased after 24 h of hypoxia (Fig. 1C). Increased p38 MAPK phosphorylation under hypoxia was confirmed by ELISA analysis (Supplemental Fig. 1D). The intracellular glucose level was not changed, but the relative amount of LDH increased (Supplemental Fig. 1E, F). The sensitivity of carfilzomib was decreased under hypoxia (Fig. 1D, Supplemental Fig. 1G). Caspase 3/7 activities also decreased after carfilzomib treatment (Fig. 1E). Nuclear factor-kappa B (NF-kB) is one of several transcription factors induced by hypoxia and cross talks with HIF1α [7]. The phosphorylation of NF-kB increased after 2 h in hypoxic culture conditions and was inhibited by the HIF1α inhibitor, FM19G11, and the p38 MAPK inhibitor, SB203580 (Fig. 1F). PFK158 is a potent and selective inhibitor of PFKFB3, and 5MPN is an inhibitor of PFKFB4, and cell proliferation was reduced (Fig. 1G, H; Supplemental Fig. 1H, I). The modest anti-proliferative action observed in U266 by carfilzomib or PFK158 may have been due to the cell line selectivity. PFK158 and 5MPN enhance carfilzomib sensitivity in hypoxic conditions (Supplemental Fig. 2A, B). The CI provides a quantitative measure of the extent of drug interactions. Because CI values were < 1, these combination treatments were synergistic (data not shown). Caspase 3/7 activity was increased, and 20S proteasome activity was reduced, by carfilzomib and PFK158 or 5MPN co-treatment; however, pro-B cell line Ba/F3 cells were not inhibited (Supplemental Fig. 2C, D, E, F). Mitochondrial membrane potential is a key indicator of mitochondrial activity [8]. The relative disrupted mitochondrial ratio was decreased, even in hypoxic conditions (Supplemental Fig. 2E). Co-treatment with carfilzomib and PFK158 or 5MPN reduced cell proliferation against the bortezomib-resistant cell line KMS-11/BTZ (Fig. 2A). Caspase 3/7 activity was also increased (Fig. 2B, C). In a previous report, BCL2L10 transgenic mice developed the characteristic features of human MM [9]. The gene expression of *BCL2L10* was increased under hypoxic conditions (Fig. 2D), and *BCL2L10* expression was correlated with *PFKFB3* and *PFKFB4* expressions (Fig. 2E). We found that the protein expressions of B-cell lymphoma 2 (BCL-2), B-cell lymphoma-extra large (BCL-XL), and BCL2L10 were reduced (Fig. 2F). Cells transfected with small hairpin RNA (shRNA) had reduced PFKFB3 or PFKFB4 expression and increased carfilzomib sensitivity (Supplemental Fig. 2H, I, J, K). PFKFB3 and PFKFB4 are cancer-specific isoenzymes [5]. Combination treatment with carfilzomib and PFK158 or 5MPN enhanced cell death by inhibition of mitochondria activity in vitro; thus, we will evaluate this inhibition in vivo in the near future. Hypoxic conditions may become an important consideration for understanding carfilzomib resistance-mediated glycolysis and NF-kB or HIF1α activation. The synergistic or sensitizing effect of PFKFB3 or PFKFB4 inhibitors with carfilzomib, suggests that these compounds could represent important adjuvants or additives in future myeloma management strategies.

**Fig. 1:**
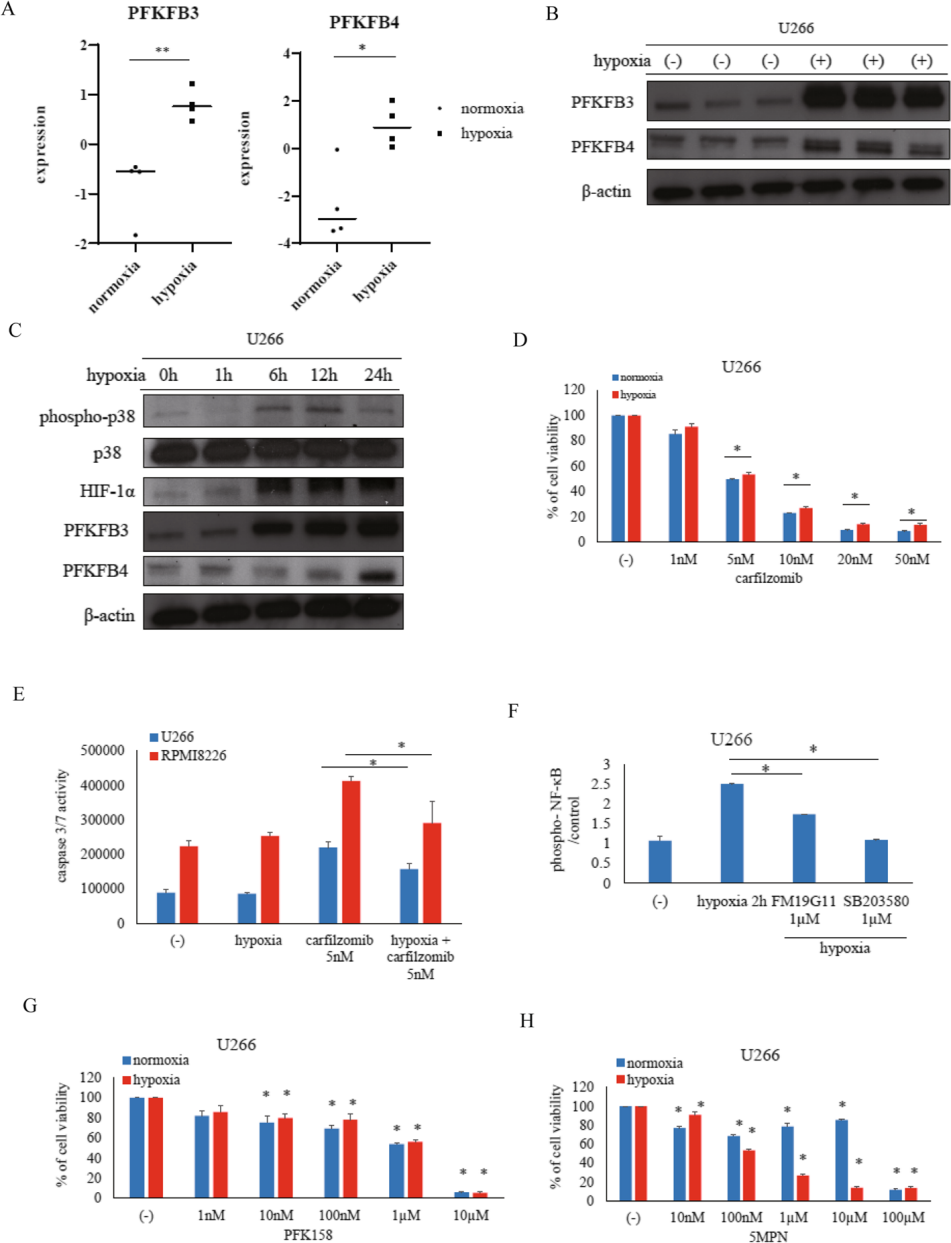
Expressions of PFKFB family members and effect of carfilzomib under normoxic and hypoxic conditions. **A** Gene expression profiles of *PFKFB* family members (*PFKFB3* and *PFKFB4*) were analyzed by comparing GEO data (GSE80140) for the normoxic (*n* = 4) and hypoxic groups (*n* = 4). *p < 0.05, **p < 0.01 vs. normoxia. n.s.: not significant. **B** Myeloma cells (U266) were cultured in RPMI 1640 medium under normoxia or hypoxia for 24 h. PFKFB3 and PFKFB4 expressions were examined by immunoblot analysis. β-actin was the loading control. Results represent the mean of three independent experiments. **C** U266 cells were cultured under hypoxia for the indicated amounts of time. Total extracts were examined by immunoblot analysis using antibodies against phospho-p38 MAPK (Thr180/Tyr182), p38 MAPK, HIF1α, PFKFB3, PFKFB4, and β-actin. **D** U266 cells were cultured under normoxia or hypoxia and incubated with the indicated concentrations of carfilzomib for 72 h. Cell growth was evaluated using Cell Counting Kit-8. *p < 0.05 vs. normoxia group. **E** U266 and RPMI8226 cells were cultured under normoxia or hypoxia and incubated with the indicated concentrations of carfilzomib for 48 h. Caspase 3/7 activity was determined using the Caspase-Glo® 3/7 Assay System. Luminescence signals were measured using the Empire Multimode Plate Reader. **F** U266 cells were cultured under normoxia or hypoxia for 2 h and incubated with the indicated concentration of FM19G11 or SB203580. Phospho- and NF-κB were analyzed using the NF-kB p65 (Phospho) [pS536] Human InstantOne™ ELISA Kit. **G**, **H** U266 cells were cultured under normoxia or hypoxia and incubated with the indicated concentrations of PFK158 or 5MPN for 72 h. Cell growth was evaluated using Cell Counting Kit-8. *p < 0.05 vs. untreated cells

**Fig. 2:**
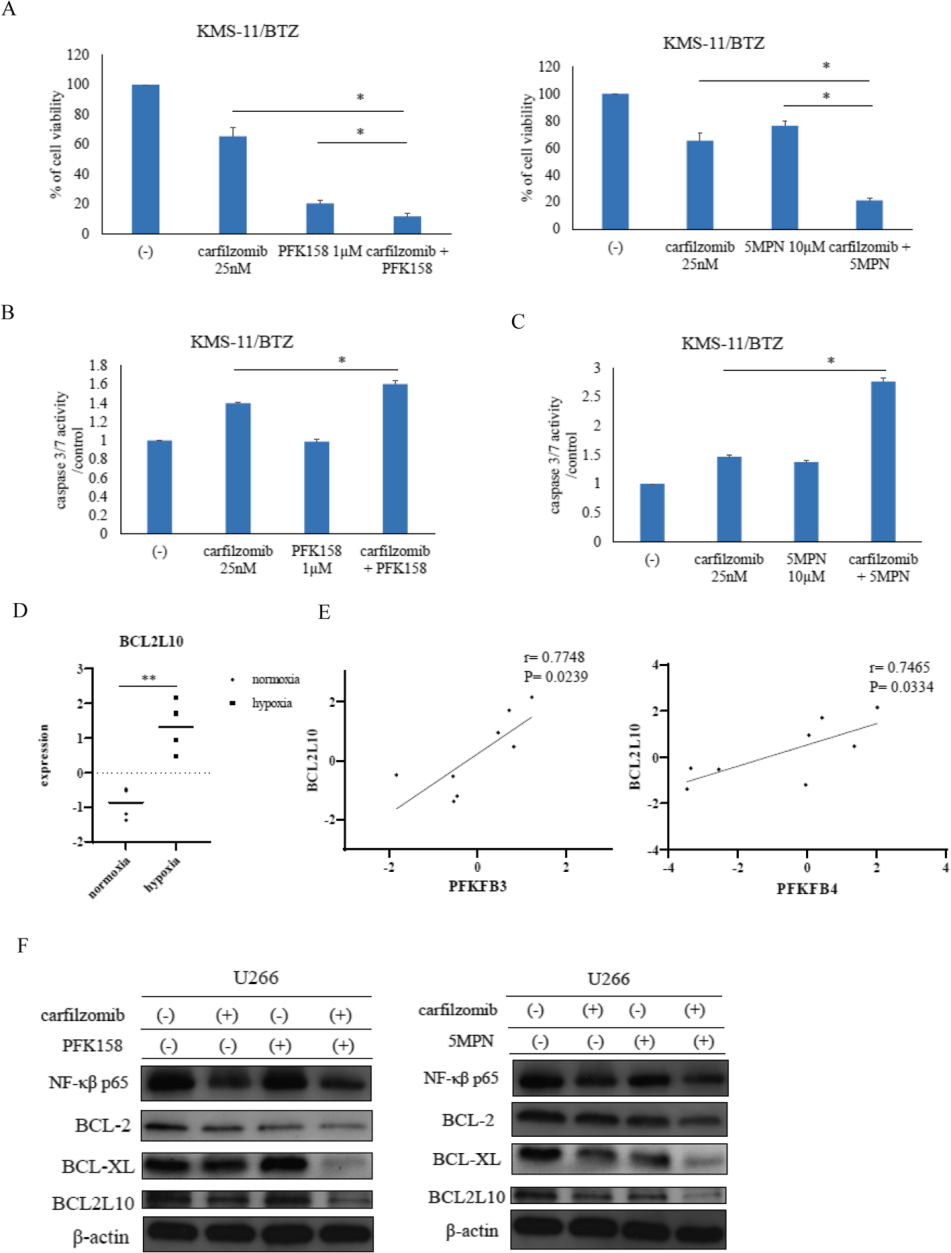
Co-treatment with carfilzomib and PFK158 or 5MPN induces cytotoxicity in myeloma cells under hypoxia. **A** KMS-11/BTZ cells were treated with carfilzomib and/or PFK158 or 5MPN for 72 h. Cell growth was evaluated using Cell Counting Kit-8. *p < 0.05 vs. carfilzomib or PFK158- or 5MPN-treated cells. **B**, **C** KMS-11/BTZ cells were treated with carfilzomib and/or PFK158 or 5MPN for 48 h. Caspase 3/7 activity was determined using the Caspase-Glo® 3/7 Assay System. *p < 0.05 vs. carfilzomib-treated cells. **D**, **E** The gene expression profile of *BCL2L10* and the correlation between *BCL2L10* and *PFKFB3* or *PFKFB4* expressions in the myeloma samples were analyzed using GEO data (GSE80140) **p < 0.01 vs. normoxia. **F** U266 cells were cultured under hypoxia for 24 h and incubated with the indicated concentrations of carfilzomib and/or PFK158 or 5MPN. Total extracts were examined by immunoblot analysis using antibodies against NF-κB p65, BCL-2, BCL-XL, BCL2L10, and β-actin

## Supplementary Information

**Additional file 1.** [10–12].

**Additional file 2: Supplemental Figure 1**. Analysis of myeloma cells under hypoxic conditions. (A) Gene expression profiles of the *PFKFB* family members (*PFKFB1* and *PFKFB2*) were analyzed by comparing GEO data (GSE80140) for the normoxic (*n* = 4) and hypoxic groups (*n* = 4). *p < 0.05, **p < 0.01 vs. normoxia. n.s.: not significant. (B, C) Myeloma cells (RPMI8226, MM.1S, MM.1R, and KMS-11/BTZ) were cultured in RPMI 1640 medium under normoxia or hypoxia for 24 h. PFKFB3 and PFKFB4 were examined using immunoblot analysis. β-actin was the loading control. Results represent the mean of three independent experiments. (D) RPMI8226 and U266 cells were cultured under normoxia or hypoxia for 6 h, and p38 MAPK activity was measured by the p38 MAPK (Phospho) [pT180/pY182] Multispecies InstantOne™ ELISA Kit. (E, F) U266 cells were cultured under normoxia or hypoxia for 24 h. Intracellular glucose and LDH release were analyzed using the Glucose Assay Kit-WST and Cytotoxicity LDH Assay kit with water-soluble tetrazolium [WST] salt. *p < 0.05 vs. normoxia or hypoxia treatment group. (G) RPMI8226 cells were cultured under normoxia or hypoxia and incubated with the indicated concentrations of carfilzomib for 72 h. Cell growth was evaluated using Cell Counting Kit-8. *p < 0.05 vs. normoxia group. (H, I) RPMI8226 cells were cultured under normoxia or hypoxia and incubated with the indicated concentrations of PFK158 or 5MPN for 72 h. Cell growth was evaluated using Cell Counting Kit-8. *p < 0.05 vs. untreated cells.

**Additional file 3: Supplemental Figure 2**. PFKFB3 and PFKFB4 inhibitors enhance the activity of proteasome inhibitors in the myeloma cell line. (A, B) U266 cells were treated with the indicated concentrations of carfilzomib and/or PFK158 (A) or 5MPN (B) for 72 h under hypoxia. Cell growth was evaluated using Cell Counting Kit-8. (C) U266 cells were treated with carfilzomib and/or PFK158 or 5MPN for 48 h. Caspase 3/7 activity was determined using the Caspase-Glo® 3/7 Assay System. *p < 0.05 vs. carfilzomib-treated cells. (D) U266 cells were treated with carfilzomib and/or PFK158 or 5MPN for 24 h. A functional assay for detecting the activity of the 20S proteasome was conducted using the 20S Proteasome Assay Kit. *p < 0.05 vs. carfilzomib-treated cells. (E) U266 cells were treated with carfilzomib and/or PFK158 or 5MPN for 24 h. Mitochondrial membrane potentials were analyzed using the cationic JC-1 dye and the Mitochondria Staining Kit. *p < 0.05 vs. carfilzomib-, PFK158-, or 5MPN-treated cells. (F) Ba/F3 cells were cultured under hypoxia and incubated with the indicated concentrations of carfilzomib for 48 h. Caspase 3/7 activity was determined using the Caspase-Glo® 3/7 Assay System. (G) Ba/F3 cells were treated with the indicated concentrations of carfilzomib and/or PFK158 or 5MPN for 72 h under normoxia or hypoxia. Cell growth was evaluated using Cell Counting Kit-8. (H) ShRNA-transfected U266 cells were cultured under normoxia or hypoxia for 24 h. Gene expressions of *PFKFB3* and *PFKFB4* were examined using quantitative RT-PCR analysis as described in the Materials and Methods. Results represent three separate experiments. (I) ShRNA-transfected U266 cells were cultured under normoxia or hypoxia for 24 h. Total extracts were examined by immunoblot analysis using antibodies against PFKFB3, PFKFB4, and β-actin. (J) ShRNA-transfected U266 cells were cultured under normoxia or hypoxia for 48 h. Caspase 3/7 activity was determined using the Caspase-Glo® 3/7 Assay System. *p < 0.05 vs. sh control cells. (K) ShRNA-transfected U266 cells were cultured under hypoxia with the indicated concentrations of carfilzomib for 72 h. Cell growth was evaluated using Cell Counting Kit-8. *p < 0.05 vs. sh control cells.

## Data Availability

Data sharing not applicable to this article as no datasets were generated or analyzed during the current study.

## Abbreviations

PFKFB: 6-phosphofructo-2-kinase/fructose-2,6-bisphosphatase
HIF1α: hypoxia-inducible factor 1α
MM: Multiple myeloma
PFK-2/FBPase-2: 6-phosphofructo-2-kinase/fructose-2,6-bisphosphatase
MGUS: Monoclonal gammopathy of undetermined significance
p38 MAPK: p38 mitogen-activated protein kinase
ATP: Adenosine triphosphate
FBS: Fetal bovine serum
RT-PCR: Real-time reverse transcription–polymerase chain reaction
GEO: Gene Expression Omnibus
NADPH: nicotinamide adenine dinucleotide phosphate
ELISA: Enzyme-linked immunosorbent assay
NF-kB: Nuclear factor-kappa beta
CI: Combination index
BCL-2: B-cell lymphoma 2
BCL-XL: B-cell lymphoma-extra large
BCL2L10: BCL-2-like protein 10
O_2_: Oxygen
CO_2_: Carbon dioxide
Abs: Antibodies
shRNA: small hairpin RNA
