# Supplementary material for: Therapeutic targeting of PFKFB3 and PFKFB4 in multiple myeloma cells under hypoxic conditions
Source: Biomark Res. 2022 May 16;10:31. doi: 10.1186/s40364-022-00376-2 (PMC9109357; doi:10.1186/s40364-022-00376-2)
Supplement: Supplementary file 1 — Additional file 1. [10–12]. [file 40364_2022_376_MOESM1_ESM.docx]

**Supplemental Methods**

**Reagents**

A potent and selective PFKFB3 inhibitor, PFK158, was purchased from MedChemExpress (Monmouth Junction, NJ, USA). A selective inhibitor of PFKFB4, 5-(n-(8-methoxy-4-quinolyl)amino)pentyl nitrate (5MPN) was purchased from Sigma–Aldrich (St. Louis, MO, USA). Carfilzomib was purchased from MedKoo Biosciences (Chapel Hill, NC, USA). Stock solutions of PFK158, 5MPN, and carfilzomib were dissolved in dimethyl sulfoxide. Other reagents were obtained from Sigma–Aldrich.

**Cell lines**

The MM cell lines, U266, RPMI8226, MM.1S, and MM.1R; mouse IL-3 dependent pro-B cell line, Ba/F3; and the murine hematopoiesis myelocytic leukemia cell line, WEHI-3B were obtained from the American Type Culture Collection (ATCC; Manassas, VA, USA). The bortezomib-resistant myeloma cell line, KMS-11/BTZ, was obtained from the Japanese Collection of Research Bioresources Cell Bank (Ibaraki, Osaka, Japan). The MM cell lines and WEHI-3B cells were cultured in Roswell Park Memorial Institute (RPMI) 1640 medium containing 10% fetal bovine serum (FBS) and 1% penicillin/streptomycin and maintained at 37°C in a 5% CO_2_-humidified atmosphere. Ba/F3 cells were cultured in RPMI 1640 medium containing 10% FBS and 10% WEHI-3B condition medium. Cell cultures were grown under hypoxic conditions at 37°C in an atmosphere of 5% CO_2_ and 1% O_2_. Experiments were initiated after 72 h of adaptation to the hypoxic conditions.

**Cell proliferation assays**

Cells were treated with PFK158 or 5MPN and/or with carfilzomib for 72 h, and then cell viability was evaluated by trypan blue exclusion or with Cell Counting Kit-8 (Dojindo Laboratories, Mashikimachi, Kumamoto, Japan) followed by measurement of the absorbance at 450 nm. In some experiments, cell cultures were also grown under hypoxic conditions (1% O_2_) and treated with PFK158 or 5MPN and/or carfilzomib. Experiments were initiated after 72 h of adaptation to the hypoxic conditions. In this study, the Chou–Talalay combination index was used to determine a synergistic combination index ((CI)<1), additive (CI=1.0), or antagonistic (CI>1) effect for each combination [10].

**Caspase 3/7 activity**

Caspase activity in myeloma cells was examined using the Caspase Glo 3/7 Assay Kit (Promega, Madison, WI, USA) according to the manufacturer’s instructions. After 48 h of carfilzomib and/or PFK158 or 5MPN treatment, the luminescence of each sample was measured using the EnSpire Multimode Plate Reader (PerkinElmer, Waltham, MA, USA).

**Enzyme-linked immunosorbent assay (ELISA)**

To investigate phospho p38 MAPK or phospho NF-κB activity, MM cells were cultured in RPMI medium with or without hypoxic conditions. After 2 or 6 h, the cells were harvested and stored at −80°C. In some experiments, the HIFα inhibitor, FM19G11 (Sigma–Aldrich), or the p38 MAPK inhibitor, SB203580 (Sigma–Aldrich), were used. P38 MAPK phosphorylation was measured using the p38 MAPK (Phospho) [pT180/pY182] Multispecies InstantOne™ ELISA Kit, and phospho NF-κB was measured using the NF-kB p65 (Phospho) [pS536] Human InstantOne™ ELISA Kit (Thermo Fisher Scientific, Waltham, MA, USA). Intracellular glucose and lactate dehydrogenase (LDH) release were analyzed using the Glucose Assay Kit-WST (Dojindo) and the LDH-Glo™ Cytotoxicity Assay (Promega) or the Cytotoxicity LDH Assay kit with water-soluble tetrazolium [WST] salt (Dojindo). The ELISA was analyzed using an EnSpire Multimode Plate Reader. All measurements were performed in triplicate.

**Proteasome activity**

The activity of the 20S proteasome was determined using the 20S Proteasome Assay Kit (Cayman Chemical, Ann Arbor, MI, USA). The cells were treated with the indicated concentrations of carfilzomib and/or PFK158 or 5MPN under hypoxia for 24 h. After the cells were harvested, the proteasome activity was analyzed according to the manufacturer’s instructions. Fluorescence intensity was measured using the EnSpire Multimode Plate Reader.

**Analysis of the mitochondrial membrane potential**

The mitochondrial membrane potential was analyzed using the cationic JC-1 dye and the Mitochondria Staining Kit (Sigma–Aldrich) according to the manufacturer’s instructions. After harvest of the MM cells, JC-1 monomers and aggregates were measured using the EnSpire Multimode Plate Reader.

**Quantitative real-time reverse transcription-polymerase chain reaction analysis (qRT-PCR)**

Total RNA was extracted from myeloma samples using the RNAqueous®-4PCR Kit (Life Technologies Japan Ltd., Minato-ku, Tokyo, Japan) and reverse transcribed using the First-Strand cDNA Synthesis Kit (OriGene Technologies, Rockville, MD, USA). RT-PCR was performed using the Roche Light Cyber 2.0 detection system (Roche Diagnostic Gmbh, Minato-ku, Tokyo, Japan). The expressions of human *PFKFB1*, *PFKFB2*, *PFKFB3*, *PFKFB4*, *GAPDH*, and *β-actin* were quantitated using the SYBR Green PCR Kit (Roche) according to the manufacturer’s protocol. Gene-specific PCR primers were obtained from Takara Bio Inc. (Otsu, Shiga, Japan).

**Immunoblot analysis**

Immunoblot analysis was performed as described previously [11,12]. After incubation, the cells were washed twice with ice-cold phosphate-buffered saline and lysed using a radioimmunoprecipitation assay lysis buffer (Merck Millipore, Darmstadt, Germany).

The protein content was quantified using the DC Protein Assay Kit (BioRad, Hercules, CA, USA) according to the manufacturer's instructions. Total cellular proteins (40 µg) were separated on 4%–20% polyacrylamide gels (BioRad) and transferred onto polyvinylidene difluoride membranes (Merck KGaA, Darmstadt, Germany). The membranes were probed using appropriate primary antibodies at the appropriate dilutions for 1 h at room temperature. The blots were visualized by chemiluminescence using the Amersham ECL chemiluminescence kit (GE Healthcare, Chicago, IL, USA). Primary antibodies (Abs) against PFKFB3 and PFKFB4 were purchased from Abcam (Cambridge, UK). NF-κB p65, cleaved caspase 3, cleaved PARP, phospho p38 MAPK, and BCL-2 Abs were purchased from Cell Signaling Technology (Danvers, MA, USA). BCL-XL, p38 MAPK, and β-actin Abs were purchased from Santa Cruz Biotechnology (Santa Cruz, CA, USA). HIF1α and BCL2L10 Abs were purchased from GeneTex (Irvine, CA, USA). Three independent experiments were performed in each case.

**shRNA transfection**

We designed and constructed an RNA interference lentiviral vector, PFKFB3- or PFKFB4-shRNA targeted against the *PFKFB3* or *PFKFB4* genes, respectively. Control shRNAs were purchased from VectorBuilder Japan, Inc. (Yokohama, Kagawa, Japan). For lentivirus infection, cells were cultured in six-well tissue culture plates and infected with the lentiviral vectors at a multiplicity of infection of 40 for 24 h in RPMI1640 medium containing 8 μg/mL hexadimethrine bromide (polybrene) (Sigma–Aldrich). The medium was replaced with fresh complete medium the following day. *PFKFB3* or *PFKFB4* expression levels before and after transfection were detected by qRT-PCR, and PFKFB3 or PFKFB4 expression levels were determined by immunoblotting.

**Statistical analysis**

All presented data were analyzed using Prism 9 (GraphPad Software, San Diego, CA, USA) or Excel software (Microsoft, Redmond, WA, USA). Student’s *t* test was used to determine if the effects on the drug-treated groups were statistically significant compared with the controls. p<0.05 or p<0.01 was considered statistically significant.
