# Supplementary figures and images for: Therapeutic targeting of PFKFB3 and PFKFB4 in multiple myeloma cells under hypoxic conditions
Source: Biomark Res. 2022 May 16;10:31. doi: 10.1186/s40364-022-00376-2 (PMC9109357; doi:10.1186/s40364-022-00376-2)

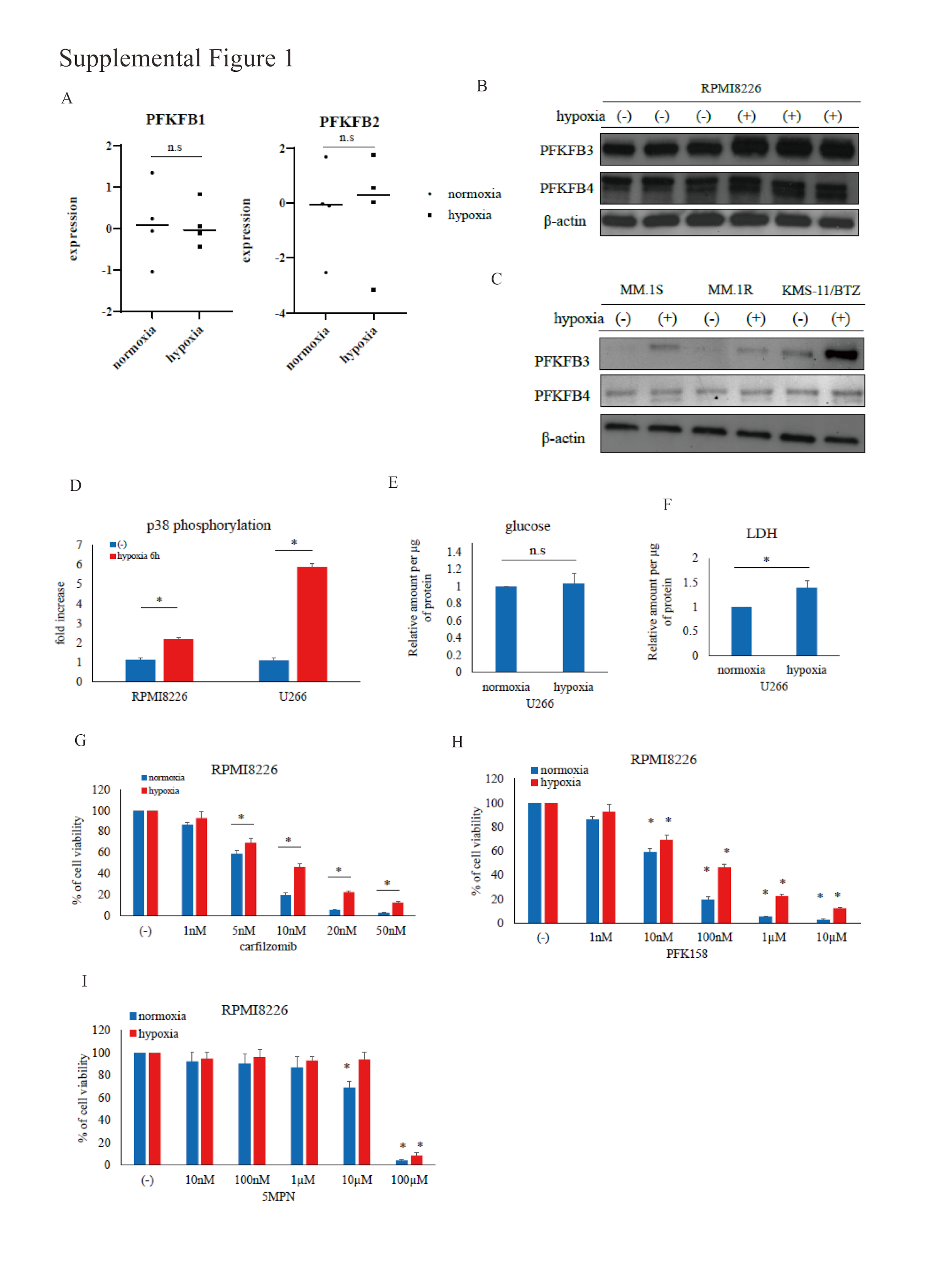

Supplement: Supplementary file 2 — Additional file 2: Supplemental Figure 1. Analysis of myeloma cells under hypoxic conditions. (A) Gene expression profiles of the PFKFB family members (PFKFB1 and PFKFB2) were analyzed by comparing GEO data (GSE80140) for the normoxic (n = 4) and hypoxic groups (n = 4). *p < 0.05, **p < 0.01 vs. normoxia. n.s.: not significant. (B, C) Myeloma cells (RPMI8226, MM.1S, MM.1R, and KMS-11/BTZ) were cultured in RPMI 1640 medium under normoxia or hypoxia for 24 h. PFKFB3 and PFKFB4 were examined using immunoblot analysis. β-actin was the loading control. Results represent the mean of three independent experiments. (D) RPMI8226 and U266 cells were cultured under normoxia or hypoxia for 6 h, and p38 MAPK activity was measured by the p38 MAPK (Phospho) [pT180/pY182] Multispecies InstantOne™ ELISA Kit. (E, F) U266 cells were cultured under normoxia or hypoxia for 24 h. Intracellular glucose and LDH release were analyzed using the Glucose Assay Kit-WST and Cytotoxicity LDH Assay kit with water-soluble tetrazolium [WST] salt. *p < 0.05 vs. normoxia or hypoxia treatment group. (G) RPMI8226 cells were cultured under normoxia or hypoxia and incubated with the indicated concentrations of carfilzomib for 72 h. Cell growth was evaluated using Cell Counting Kit-8. *p < 0.05 vs. normoxia group. (H, I) RPMI8226 cells were cultured under normoxia or hypoxia and incubated with the indicated concentrations of PFK158 or 5MPN for 72 h. Cell growth was evaluated using Cell Counting Kit-8. *p < 0.05 vs. untreated cells. [file 40364_2022_376_MOESM2_ESM.tif]

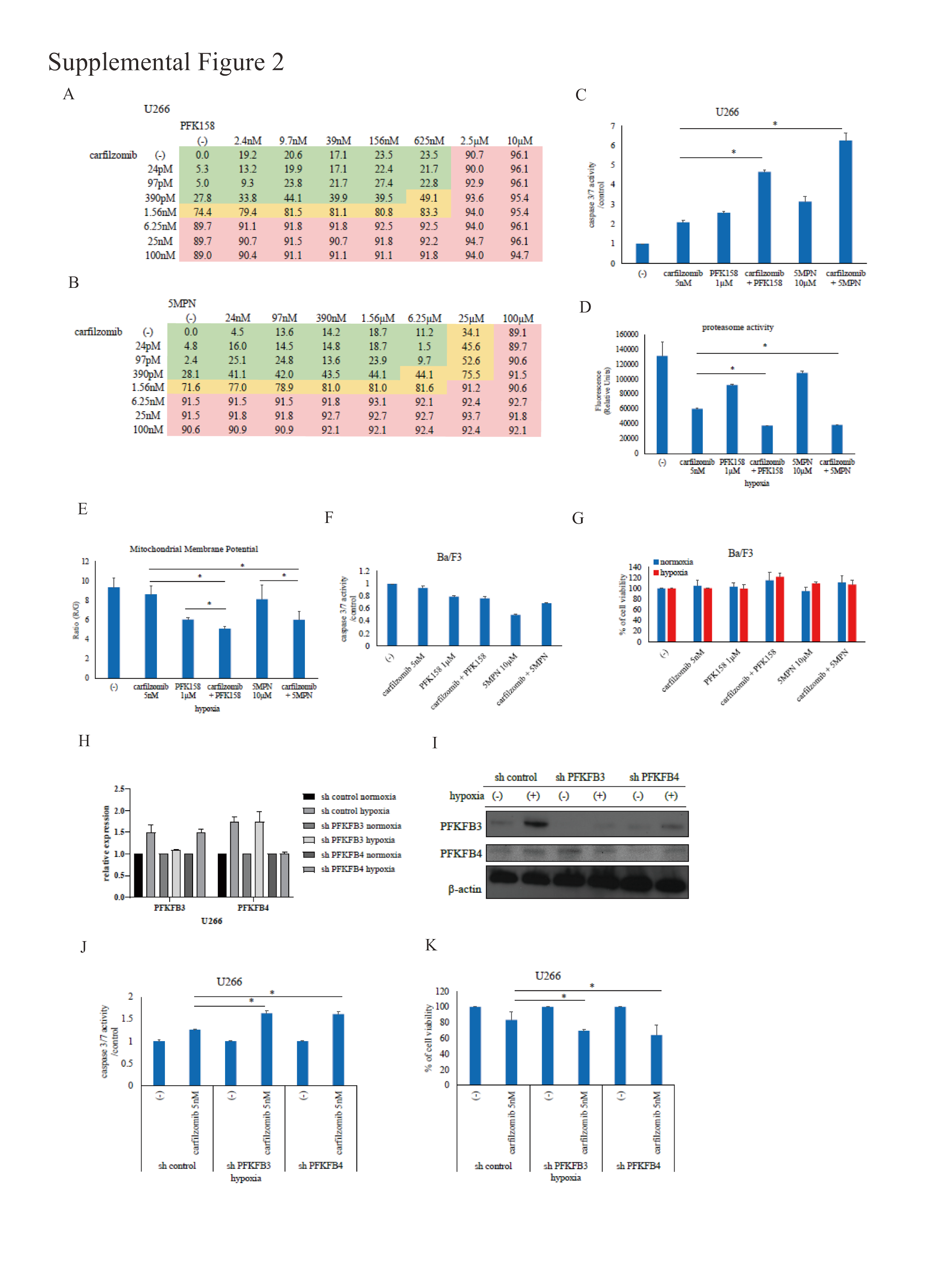

Supplement: Supplementary file 3 — Additional file 3: Supplemental Figure 2. PFKFB3 and PFKFB4 inhibitors enhance the activity of proteasome inhibitors in the myeloma cell line. (A, B) U266 cells were treated with the indicated concentrations of carfilzomib and/or PFK158 (A) or 5MPN (B) for 72 h under hypoxia. Cell growth was evaluated using Cell Counting Kit-8. (C) U266 cells were treated with carfilzomib and/or PFK158 or 5MPN for 48 h. Caspase 3/7 activity was determined using the Caspase-Glo® 3/7 Assay System. *p < 0.05 vs. carfilzomib-treated cells. (D) U266 cells were treated with carfilzomib and/or PFK158 or 5MPN for 24 h. A functional assay for detecting the activity of the 20S proteasome was conducted using the 20S Proteasome Assay Kit. *p < 0.05 vs. carfilzomib-treated cells. (E) U266 cells were treated with carfilzomib and/or PFK158 or 5MPN for 24 h. Mitochondrial membrane potentials were analyzed using the cationic JC-1 dye and the Mitochondria Staining Kit. *p < 0.05 vs. carfilzomib-, PFK158-, or 5MPN-treated cells. (F) Ba/F3 cells were cultured under hypoxia and incubated with the indicated concentrations of carfilzomib for 48 h. Caspase 3/7 activity was determined using the Caspase-Glo® 3/7 Assay System. (G) Ba/F3 cells were treated with the indicated concentrations of carfilzomib and/or PFK158 or 5MPN for 72 h under normoxia or hypoxia. Cell growth was evaluated using Cell Counting Kit-8. (H) ShRNA-transfected U266 cells were cultured under normoxia or hypoxia for 24 h. Gene expressions of PFKFB3 and PFKFB4 were examined using quantitative RT-PCR analysis as described in the Materials and Methods. Results represent three separate experiments. (I) ShRNA-transfected U266 cells were cultured under normoxia or hypoxia for 24 h. Total extracts were examined by immunoblot analysis using antibodies against PFKFB3, PFKFB4, and β-actin. (J) ShRNA-transfected U266 cells were cultured under normoxia or hypoxia for 48 h. Caspase 3/7 activity was determined using the Caspase-Glo® 3/7 Assay System. [file 40364_2022_376_MOESM3_ESM.tif]
